# A Novel Growth-Based Selection Strategy Identifies New Constitutively Active Variants of the Major Virulence Regulator PrfA in Listeria monocytogenes

**DOI:** 10.1128/JB.00115-20

**Published:** 2020-05-11

**Authors:** Sabine Hansen, Michael Hall, Christin Grundström, Kristoffer Brännström, A. Elisabeth Sauer-Eriksson, Jörgen Johansson

**Affiliations:** a Department of Molecular Biology, Umeå University, Umeå, Sweden; b Department of Chemistry, Umeå University, Umeå, Sweden; c Umeå Center for Microbial Research (UCMR), Umeå University, Umeå, Sweden; d Department of Medical Biochemistry and Biophysics, Umeå University, Umeå, Sweden; e Molecular Infection Medicine, Sweden (MIMS), Umeå University, Umeå, Sweden; University of Illinois at Chicago

**Keywords:** *Listeria monocytogenes*, PrfA, PrfA*, crystal structure, LLO, ActA

## Abstract

Listeria monocytogenes is a Gram-positive pathogen able to cause severe human infections. Its major virulence regulator is the transcriptional activator PrfA, a member of the Crp/Fnr family of transcriptional regulators. To establish a successful L. monocytogenes infection, the PrfA protein needs to be in an active conformation, either by binding the cognate inducer glutathione (GSH) or by possessing amino acid substitutions rendering the protein constitutively active (PrfA*). By a yet unknown mechanism, phosphotransferase system (PTS) sugars repress the activity of PrfA. We therefore took a transposon-based approach to identify the mechanism by which PTS sugars repress PrfA activity. For this, we screened a transposon mutant bank to identify clones able to grow in the presence of glucose-6-phosphate as the sole carbon source. Surprisingly, most of the isolated transposon mutants also carried amino acid substitutions in PrfA. In transposon-free strains, the PrfA amino acid substitution mutants displayed growth, virulence factor expression, infectivity, and DNA binding, agreeing with previously identified PrfA* mutants. Hence, the initial growth phenotype observed in the isolated clone was due to the amino acid substitution in PrfA and unrelated to the loci inactivated by the transposon mutant. Finally, we provide structural evidence for the existence of an intermediately activated PrfA state, which gives new insights into PrfA protein activation.

**IMPORTANCE** The Gram-positive bacterium Listeria monocytogenes is a human pathogen affecting mainly the elderly, immunocompromised people, and pregnant women. It can lead to meningoencephalitis, septicemia, and abortion. The major virulence regulator in L. monocytogenes is the PrfA protein, a transcriptional activator. Using a growth-based selection strategy, we identified mutations in the PrfA protein leading to constitutively active virulence factor expression. We provide structural evidence for the existence of an intermediately activated PrfA state, which gives new insights into PrfA protein activation.

## Abstract (precis)

The Gram-positive bacterium Listeria monocytogenes is a human pathogen affecting mainly the elderly, immunocompromised people, and pregnant women. It can lead to meningoencephalitis, septicemia, and abortion. The major virulence regulator in L. monocytogenes is the PrfA protein, a transcriptional activator. Using a growth-based selection strategy, we identified mutations in the PrfA protein leading to constitutively active virulence factor expression. We provide structural evidence for the existence of an intermediately activated PrfA state, which gives new insights into PrfA protein activation.

## INTRODUCTION

Listeria monocytogenes is a Gram-positive bacterium that naturally resides in the soil. Occasionally, L. monocytogenes can become a human pathogen upon ingestion. The elderly, immunocompromised people, and pregnant women are at risk since the bacterium can cause meningoencephalitis, septicemia, and abortion (1–3). To cause an infection in the human host, L. monocytogenes conducts the expression and action of an arsenal of virulence and host factors. Invasion of different cell types requires the expression of various surface proteins such as internalins (InlA and InlB) and actin assembly-inducing protein (ActA) (4). Once inside the cell, the bacterium is trapped in membrane-bound vacuoles that are lysed upon expression and secretion of bacterial proteins such as listeriolysin O (LLO) and two phospholipases (PlcA and PlcB) (1). After escape from the vacuole, the bacteria enter the cytosol, where they start expressing the hexose-phosphate transporter Hpt. This enables the bacteria to make use of the sugar sources available inside the mammalian cell, thereby allowing bacterial replication (5). Inside the cytosol, the bacteria also start expressing the surface protein ActA, which allows them to move through the cell and into adjacent ones, using host cell actin polymerization for motility (6–8).

The major regulator of virulence factors in L. monocytogenes is the transcription activator PrfA, a member of the Crp/Fnr family of regulators. Outside the host, the expression of PrfA-regulated genes is low, but upon entering a host, PrfA becomes activated and turns on the expression of PrfA-regulated virulence genes. For activation, PrfA requires binding of the cofactor glutathione (9, 10). Glutathione binding stabilizes the DNA-binding helix-turn-helix (HTH) motif in a conformation compatible with DNA-binding, thereby allowing expression of PrfA-regulated virulence factors (10). The expression of virulence factors is tightly regulated to prevent their expression when they are not needed. How the bacterium controls this virulence factor expression is still not fully understood. It is known that PrfA-regulated gene products are repressed when the bacterium is grown in broth containing phosphoenolpyruvate phosphotransferase system (PTS) sugars such as cellobiose and glucose (reviewed in references 1 and [Bibr B11][Bibr B12][Bibr B13]). However, when grown in LB medium supplemented with non-PTS sugars carrying a phosphate group (i.e., sugar phosphates such as glucose-1-phosphate [G-1-P], glucose-6-phosphate [G-6-P], mannose-6-phosphate [M-6-P], and fructose-6-phosphate [F-6-P]), there is no repression of virulence gene expression (5, 14). Since sugar phosphates, unlike glucose and cellobiose, are taken up by the Hpt transporter and not by the PTS, it has been suggested that an active PTS represses PrfA activity, although the mechanism remains unclear. This would repress PrfA activity when the bacterium lives in the soil, where its primary sugar sources are PTS sugars (5). In contrast, PrfA becomes active once inside the mammalian host, where the sugar sources are available in the form of sugar phosphate.

It was previously shown that sugar-phosphate utilization is strictly dependent on PrfA activity; an L. monocytogenes strain carrying a glycine-to-serine substitution at position 145 (PrfA_G145S_) renders the protein constitutively active. In contrast to a bacterial strain carrying wild-type PrfA (PrfA_WT_), this PrfA_G145S_ mutant is able to metabolize G-1-P (5, 14). Several other amino acid substitutions resulting in active PrfA proteins (called PrfA*) have also been identified (reviewed in reference 15). These mutant strains are all characterized by elevated PrfA-dependent gene expression under nonvirulence conditions, such as growth in the presence of PTS sugars. Different PrfA* mutants are able to activate virulence gene expression to various extents.

Previous data show that L. monocytogenes is unable to grow in chemically defined medium (DM) with sugar phosphate as the sole carbon source (16). We found that L. monocytogenes PrfA* mutants can grow in DM supplemented with G-6-P and that this growth phenotype is strictly dependent on high expression of Hpt. Since growth of L. monocytogenes in G-6-P requires an active version of PrfA, we screened a transposon mutant library with the aim of identifying genes involved in sugar-mediated repression of PrfA activity. Surprisingly, for most of the isolated mutants, their ability to grow in the G-6-P medium was due to amino acid substitutions in PrfA, rendering them PrfA*. We identified three previously unidentified PrfA* variants of different classes that we characterized on the basis of their virulence factor expression, infectivity, and DNA binding. Structure analyses show that the newly isolated PrfA* protein dimers fold into intermediate structures, i.e., without a collapsed central structure, with one HTH motif in an unstructured inactive form and one HTH motif in an active folded form. We refer to these structures as intermediate-active forms of PrfA. Combined with previous work (17), this research shows structural evidence that PrfA can exist in at least three forms of activation—inactive, intermediately active, and fully active.

## RESULTS

### Growth of Listeria monocytogenes in defined medium supplemented with G-6-P requires a constitutively active PrfA protein.

**FIG 1:**
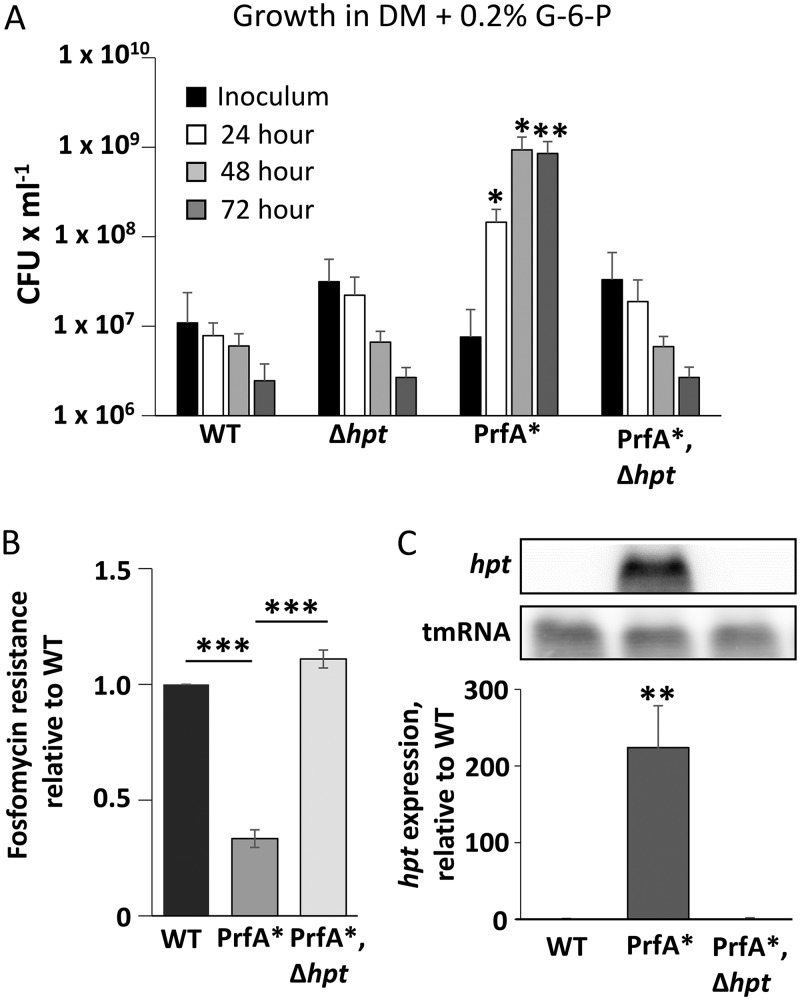
Growth of Listeria monocytogenes in defined medium (DM) supplemented with glucose-6-phosphate (G-6-P). (A) Four strains were tested for growth in DM with 0.2% G-6-P as the sole carbon source. These were (i) the wild-type EGDe strain (WT), (ii) the strain carrying an in-frame deletion of the *hpt* gene (Δ*hpt*), (iii) the strain carrying the Gly to Ser substitution of codon 145 in the PrfA protein (PrfA_G145S_), and (iv) the PrfA_G145S_ strain carrying an in-frame deletion of the *hpt* gene (PrfA_G145S_ Δ*hpt*). Growth was monitored by viable count for 3 days and shown as mean values with standard deviations (*n* = 3). Statistical analysis was used to compare growth of the mutant strains with that of the wild-type strain at each time point (Student’s *t* test [two tailed; *, *P* < 0.05; **, *P* < 0.01]). (B) Fosfomycin resistance of the indicated strains. Fosfomycin discs were used, and the clearing zone was measured. The radius of the clearing zone is indicated relative to the wild type as an average of three independent experiments. Statistical analysis was used to compare fosfomycin sensitivity of the PrfA* strain with the WT and the PrfA* and Δ*hpt* strains (Student’s *t* test [two tailed; ***, *P* < 0.001]). (C) (Top) Expression of *hpt* in the indicated strains grown in BHI until the OD_600_ is 1. RNA was isolated and *hpt* expression was examined with Northern blotting using radiolabeled probes against *hpt* and tmRNA (control). A representative of three independent experiments is shown. (Bottom) Measurement of *hpt* expression from the top panel. Expression is relative to WT (set to 1). Student’s *t* test (two tailed; ***, *P* < 0.001).

Growth of L. monocytogenes in the presence of sugar phosphate requires the hexose phosphate transporter Hpt (5). Hpt is expressed once the bacterium enters the host cytosol, where Hpt expression and sugar phosphate uptake require functional PrfA (5). In line with these findings, we tested if a constitutively active form of PrfA, PrfA*, could grow in defined medium (DM) with sugar phosphate as the sole carbon source. To test this hypothesis and evaluate it as a selection strategy for identifying genes involved in PTS sugar-mediated repression of PrfA activity, four strains were plated on DM supplemented with G-6-P. The strains tested were a wild-type strain (WT), a strain lacking Hpt (Δ*hpt*), a PrfA* strain (*prfA_G145S_*), and the same PrfA* strain lacking Hpt (*prfA_G145S_*, Δ*hpt*) (Fig. 1A). The PrfA_G145S_ mutant strain grew on the DM/G-6-P medium, in contrast to the WT and Δ*hpt* mutant strains. The growth of the PrfA_G145S_ mutant was clearly dependent on Hpt, as there was no growth of the PrfA_G145S_ Δ*hpt* mutant (Fig. 1A).

Next, we tested the fosfomycin sensitivity of the WT, *prfA_G145S_*, and *prfA_G145S_*, Δ*hpt* strains, as fosfomycin sensitivity correlates directly with the level of Hpt expression (18). As expected, the PrfA_G145S_ mutant strain showed increased fosfomycin sensitivity compared to the WT strain (Fig. 1B). Furthermore, the increased fosfomycin sensitivity was completely abolished in the PrfA_G145S_ Δ*hpt* strain. Since PrfA positively controls *hpt* expression (5, 19), we examined *hpt* expression in the WT and the two PrfA_G145S_ mutant strains. All strains were grown in nutrient-rich brain heart infusion (BHI) broth prior to RNA isolation and Northern blot analysis (Fig. 1C). In agreement with the hypothesis that *hpt* expression requires active PrfA, only the PrfA_G145S_ mutant strain could express *hpt*. Taken together, these results suggest that growth of L. monocytogenes on G-6-P as the sole carbon source could be used as a selection strategy to better understand mechanisms of sugar-mediated repression of PrfA activity.

### Isolation of constitutively active PrfA while screening a transposon mutant library.

To identify genes involved in sugar-mediated repression of PrfA activity, we tested a previously generated *Himar1-mariner* transposon (Tn) mutant library for growth in DM medium supplemented with G-6-P as the sole carbon source (20). If the Tn mutants grew with G-6-P as the sole carbon source, we hypothesized that there were four possible explanations: (i) the mutant strain had to carry an activated PrfA due to a Tn insertion in a gene encoding a protein involved in PrfA inhibition; (ii) the Tn was inserted in a gene that acts as a repressor of Hpt expression in a PrfA-independent manner; (iii) the mutant strain carried a transposon-independent mutation in the *prfA* gene that makes the PrfA protein constitutively active; or (iv) the mutant strain carried a transposon-independent mutation in the *hpt* loci, making Hpt constitutively expressed.

The screen was conducted as described in Fig. S1 in the supplemental material and Materials and Methods. Briefly, a library of individual transposon mutants (*n* = 13,344) was inoculated in BHI overnight before reinoculation in DM supplemented with 0.2% (wt/vol) G-6-P and was tested for growth over several days. Of the 13,344 transposon mutants screened, 19 grew after 3 days in DM supplemented with G-6-P (corresponding to 0.14% of the tested colonies). As we were interested in identifying mutants with increased PrfA activity, the 19 mutants were plated on blood agar plates for hemolytic activity as a readout for PrfA activity. Of the 19 mutants, 12 had higher hemolytic activity than the WT control and were selected for further studies.

Before further characterization of transposon insertions, we sequenced the *prfA* gene of the 12 isolated mutants. We wanted to examine if any of them had acquired a transposon-independent mutation in the *prfA* gene, rendering them constitutively active (PrfA*). Surprisingly, we found that most isolated transposon mutants (10 out of 12) also carried a point mutation in the *prfA* gene, leading to amino acid substitutions in the protein. The two remaining mutants did not have base substitutions in the *prfA* gene and are currently undergoing further analysis in our laboratory. The *prfA* gene modifications included three previously characterized amino acid substitutions (1, 21, 22) as follows: four of the ten mutants carried the Gly to Ser substitution at residue 145 (PrfA_G145S_), one carried the Gly to Cys substitution at residue 145 (PrfA_G145C_), and two carried the Leu to Phe substitution at residue 140 (PrfA_L140F_). In addition, we found three new PrfA mutants in the remaining three strains—Leu to His substitution at residue 140 (PrfA_L140H_), Ala to Gly substitution at residue 218 (PrfA_A218G_), and Ala to Val substitution at residue 94 (PrfA_A94V_). Previously, the Ala to Thr substitution at residue 94 (PrfA_A94T_) was shown to give rise to a PrfA* phenotype, although this substitution has not been extensively characterized (22).

### Analysis of the identified PrfA mutants.

**FIG 2:**
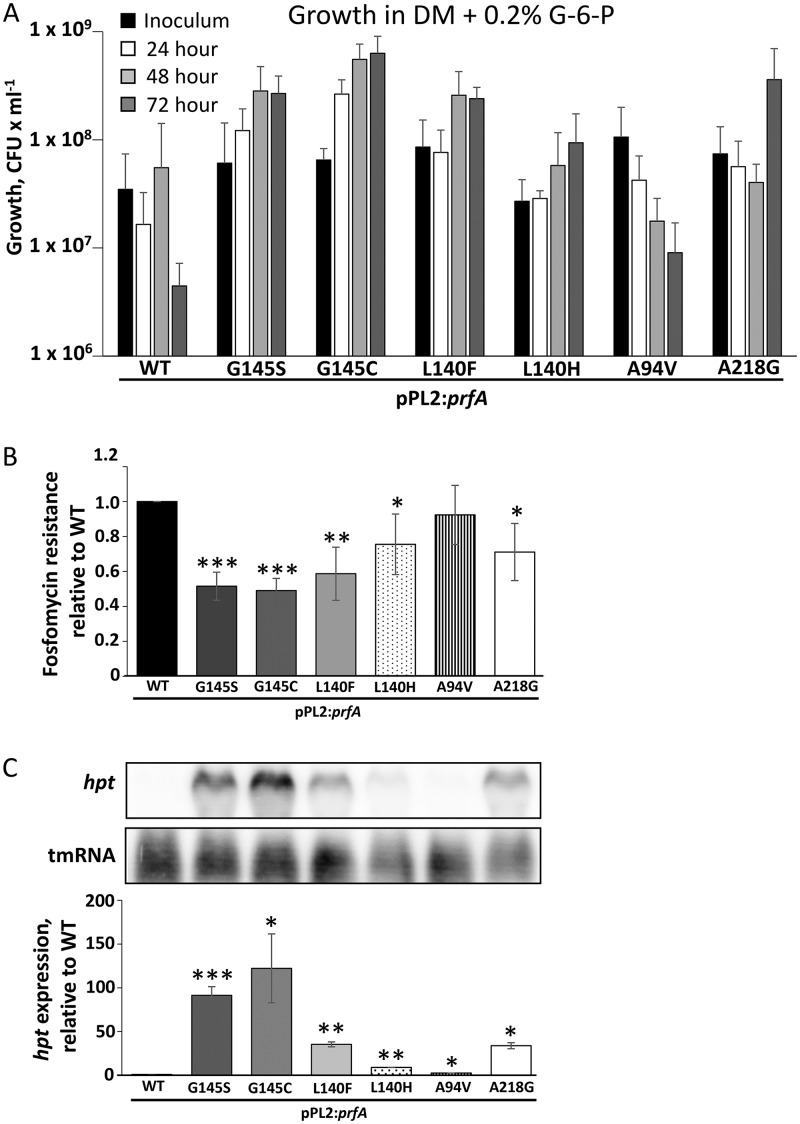
Effect of the amino acid substitutions on PrfA activity. (A) Transposon-free strains expressing PrfA proteins with the wild-type sequence or indicated amino acid substitutions were tested for growth in DM supplemented with 0.2% G-6-P for 72 h. A representative of five independent experiments is shown. (B) Fosfomycin resistance of the indicated strains. Fosfomycin discs were used, and the clearing zone was measured. The radius of the clearing zone is indicated relative to the wild type as an average of three independent experiments. Statistical analysis was used to compare fosfomycin sensitivity of the PrfA* strain with the that of the wild type and the PrfA* and Δ*hpt* strains (Student’s *t* test [two tailed; *, *P* < 0.05; **, *P* < 0.01; ***, *P* < 0.001]). (C) (Top) Expression of *hpt* in the indicated strains grown in BHI until the OD_600_ reached 1. RNA was isolated and *hpt* expression was examined with Northern blotting using radiolabeled probes against *hpt* and tmRNA (control). A representative of three independent experiments is shown. (Bottom) Quantification of *hpt* expression from the top panel. Expression is relative to the WT (set to 1). Student’s *t* test (two tailed; *, *P* < 0.05; **, *P* < 0.01; ***, *P* < 0.001).

Our results indicated that the phenotypes of the mutants (growth in DM supplemented with G-6-P, and hemolytic activity) were due to mutations in the *prfA* gene. However, we needed to rule out the possibility that the transposons or other secondary site mutations were affecting the PrfA activity. L. monocytogenes strains carrying the *prfA* mutations were therefore constructed in the integrative pPL2 plasmid in a genetic background devoid of transposons (23). To avoid variations in PrfA protein levels, the *prfA*_wt_ strain together with the *prfA* mutants were introduced in the pPL2 plasmid carrying the *prfAp_1_* and *prfAp_2_* promoters. At the same time, the PrfA-controlled *plcA* promoter was omitted to avoid unwanted indirect effects of the positive PrfA feedback loop. The WT and the mutant versions of *prfA* were introduced as a single copy into the strain EGDe (WT) containing an in-frame deletion of the *prfA* gene (Δ*prfA*). None of the PrfA mutant strains showed any growth defect at 37°C when grown in BHI, in agreement with previous observations (Fig. S2) (22). The mutant strains were further tested for growth in DM supplemented with G-6-P (Fig. 2A). All the previously identified PrfA* mutants, i.e., PrfA_G145S_, PrfA_G145C_, and PrfA_L140F_, could grow in this medium with G-6-P, as could the PrfA mutants PrfA_L140H_ and PrfA_A218G_, whereas the strain carrying the PrfA_A94V_ mutation was unable to grow. As growth in G-6-P is completely dependent on Hpt expression, we tested the fosfomycin sensitivity of the mutants. All the mutants exhibited significantly increased fosfomycin sensitivity compared to the PrfA_WT_ strain, with the A94V mutant showing the least sensitivity (Fig. 2B). We next tested *hpt* expression using Northern blots. The results were consistent with the fosfomycin sensitivity test—all the *prfA* mutant strains showed significantly increased *hpt* expression compared to the wild-type strain, with the A94V substitution having a minute increase in *hpt* expression (Fig. 2C). Based on these data, we hypothesize that the original A94V transposon mutant carried a secondary site mutation, allowing it to grow in medium having G-6-P as the sole carbon source. In view of this, for this study we decided not to pursue this A94V strain further.

### Characterization of the PrfA* phenotype.

**FIG 3:**
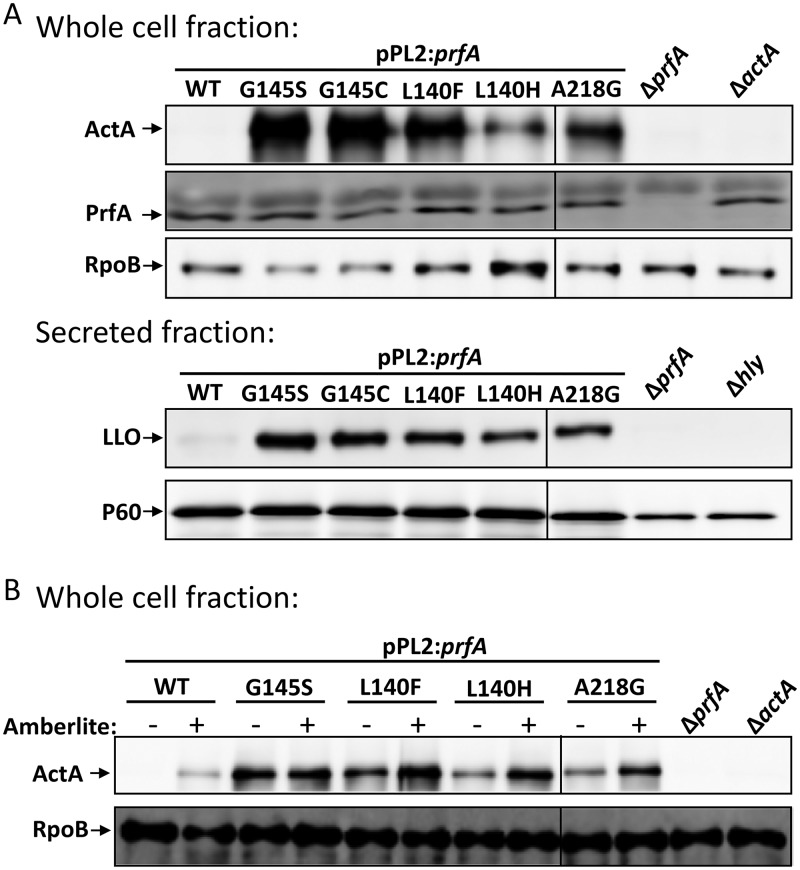
The identified point mutations in PrfA give rise to PrfA* phenotypes. (A) ActA, PrfA, and LLO virulence factor expression of the indicated strains examined using Western blots. The strains were grown in BHI until the OD_600_ reached 1. RNA polymerase beta (RpoB) was used as a loading control for whole-cell fraction samples (ActA and PrfA), and P60 was used as a loading control for the secreted fraction (LLO). A representative of four independent experiments is shown. (B) Expression of ActA and PrfA virulence factors in the presence of 1% Amberlite. The strains were grown in BHI with or without 1% Amberlite XAD4 until the OD_600_ reached 1 before sample preparation and Western blotting. RNA polymerase beta (RpoB) was used as a loading control. A representative of four independent experiments is shown. See also Fig. S3 for quantification of ActA, PrfA, and LLO expression levels from panel A and Fig. S4 for quantification of ActA expression levels from panel B.

To further characterize the isolated *prfA* base substitution mutants, we examined virulence factor expression using Western blots. ActA and LLO protein levels were upregulated in all the *prfA* mutant strains compared to wild-type levels (Fig. 3A and Fig. S3). The PrfA_G145S_, PrfA_G145C_, and PrfA_L140F_ mutants showed the highest expression levels, followed by PrfA_A218G_ and PrfA_L140H_. Importantly, no significant differences in the amount of PrfA were observed among the strains. This indicates that the effect on the virulence gene expression was due to increased PrfA activity and not to increased PrfA expression (24). Based on the growth in DM supplemented with G-6-P, *hpt* expression, fosfomycin resistance, and virulence factor expression, all the PrfA mutants exhibited PrfA* phenotypes, albeit to various degrees.

L. monocytogenes grown in BHI medium supplemented either with charcoal or with the nonpolar adsorbent Amberlite XAD4 have increased virulence gene expression, presumably due to the removal (by the charcoal/Amberlite) of a hitherto unidentified inhibitory substance released by L. monocytogenes during growth (24). Amberlite XAD4 is a polymeric adsorbent known to be especially effective against low-molecular-weight hydrophobic compounds (24). When grown in BHI supplemented with Amberlite XAD4 (BHIA), the wild-type and PrfA_L140F_ mutant strains showed significantly increased expression of ActA compared to bacteria grown in only the BHI (Fig. 3B, Fig. S4). Also, the PrfA_L140H_ and the PrfA_A218G_ show induced ActA expression despite not being statistically significant. Together, our data suggest that PrfA_WT_, PrfA_L140F_ PrfA_L140H_, and PrfA_A218G_ but not PrfA_G145C_ can be further activated.

### Intracellular phenotype of the PrfA* mutants.

**FIG 4:**
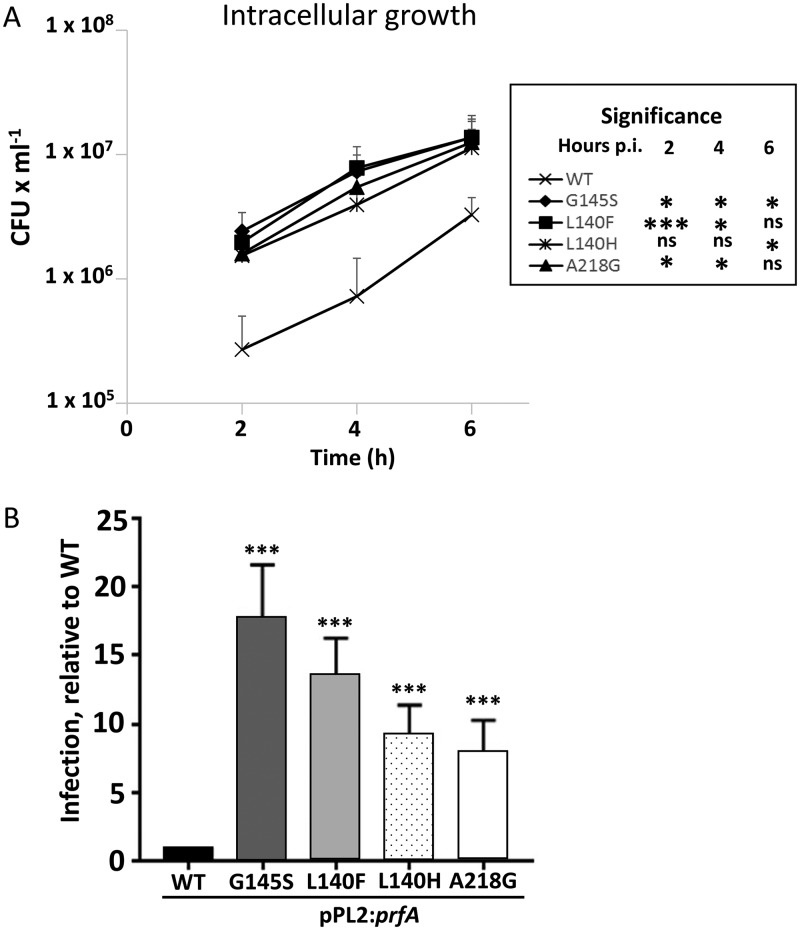
The PrfA* mutant strains show a larger uptake and cell-to-cell spread compared to a wild-type strain. (A) Intracellular growth of the indicated strains was tested by viable count in the colon epithelial cell line Caco-2. An average with standard deviation of three independent experiments is shown. Statistical analysis (inset) compared the number of CFU for the wild-type strain with that of the mutant strains at 2, 4, and 6 h postinfection (Student’s *t* test, [two tailed; *, *P* < 0.05; ***, *P* < 0.001; ns, no significant difference]). (B) The indicated strains were tested for infection (adhesion/invasion and/or cell-to-cell spread) of the Caco-2 derivative TC7 cell line using a multiplicity of infection of 1:500. The number of plaques formed was determined and represented as the number of plaques relative to the WT (set to 1). An average with standard deviation from three independent experiments is shown. Statistical analysis compared infection of the wild-type strain with that of the mutant strains (Student’s *t* test [two tailed; **, *P* < 0.01; ***, *P* < 0.001]).

The phenotype of the identified mutants was investigated in more detail. We started by investigating the intracellular growth of the mutant strains in the colon epithelial cell line Caco-2 (Fig. 4A). At 2 h postinfection, all tested mutants displayed a greater number of intracellular bacteria than the wild-type strain. However, the intracellular growth rates of the mutant strains were not markedly increased compared to the wild-type strain, suggesting that PrfA_WT_ is fully activated 2 h postinfection. During infection, an important feature of L. monocytogenes pathogenesis is its capacity to spread from cell to cell. This can be monitored by plaque formation in monolayers of tissue culture cells, which correlates well with the virulence seen in a mouse model (25). As a complementary strategy to further examine the ability of our strains to infect cells, we employed a modified plaque assay, which qualitatively assessed the ability of bacteria to adhere, invade, and/or spread from cell to cell. We counted the number of plaques formed and correlated that with the wild type (Fig. 4B). The mutants all showed an increased ability to form plaques in a TC7 cell line compared to the wild type. In summary, the different PrfA* variants gave rise to diverse levels of PrfA activity, with PrfA_G145S_ being almost fully activated and the other mutants showing lower activity.

### Differential DNA-binding capacity among the PrfA* proteins.

Previous studies with electrophoresis mobility shift assay or surface plasmon resonance (SPR) demonstrate that the PrfA_G145S_ protein has a higher binding affinity than the PrfA_WT_ protein for the *hly* and *actA* DNA promoters (17). Here, we show that all the PrfA* proteins identified in our screen had higher binding affinities to *hly*, *actA*, and *hpt* promoter sequences than the PrfA_WT_ protein (Table 1). The previously identified PrfA_G145S_ protein showed the lowest equilibrium binding constant, i.e., strongest binding of all the mutants, followed by the PrfA_A218G_, PrfA_L140H_, and PrfA_L140F_ proteins, respectively.

**TABLE 1:** SPR analysis of association rate constant (*k_a_*), dissociation rate constant (*k_d_*), and dissociation constant (*K_D_*)

Protein	Data for *hly*			Data for *actA*			Data for *hpt*		
	*k_a_* (M^−1^ s^−1^)	*k_d_* (s^−1^)	*K_D_* (nM)	*k_a_* (M^−1^ s^−1^)	*k_d_* (s^−1^)	*K_D_* (nM)	*k_a_* (M^−1^ s^−1^)	*k_d_* (s^−1^)	*K_D_* (nM)
PrfA_WT_	1.7 · 10^5^	1.9 · 10^–2^	110	6.2 · 10^4^	4.2 · 10^–2^	690	4.2 · 10^4^	5.9 · 10^–2^	1040
PrfA_L140F_	1.7 · 10^5^	2.5 · 10^–3^	15	9.2 · 10^4^	8.5 · 10^-3^	95	7.0 · 10^4^	1.9 · 10^–2^	270
PrfA_L140H_	6.6 · 10^5^	6.8 · 10^–3^	10	3.5 · 10^5^	3.0 · 10^–2^	85	2.6 · 10^5^	5.4 · 10^–2^	210
PrfA_A218G_	1.6 · 10^6^	6.4 · 10^–3^	4	8.6 · 10^5^	2.3 · 10^–2^	27	7.1 · 10^5^	3.9 · 10^–2^	55
PrfA_G145S_	1.9 · 10^6^	3.8 · 10^–3^	2	4.0 · 10^5^	5.0 · 10^–3^	12	2.5 · 10^5^	7.7 · 10^–3^	31

### Structural organization of PrfA* homodimers.

To gain a deeper understanding of the PrfA* mutant proteins, the crystal structures of purified PrfA_L140H_, PrfA_L140F_, and PrfA_A218G_ were determined as individual proteins and in complex with the PrfA *hly* promoter DNA (Table S1). These structures were compared to the known structures of PrfA_WT_ (PDB codes 2BEO [17] and 5F1R [19]), PrfA_G145S_ (PDB code 2BGC [17]), the glutathione-activated PrfA (PrfA_WT_-GSH; PDB code 5LRR [10]), and PrfA in complex with promoter *hly* DNA (PrfA_WT_-DNA; PDB code 5LEJ [10]).

**FIG 5:**
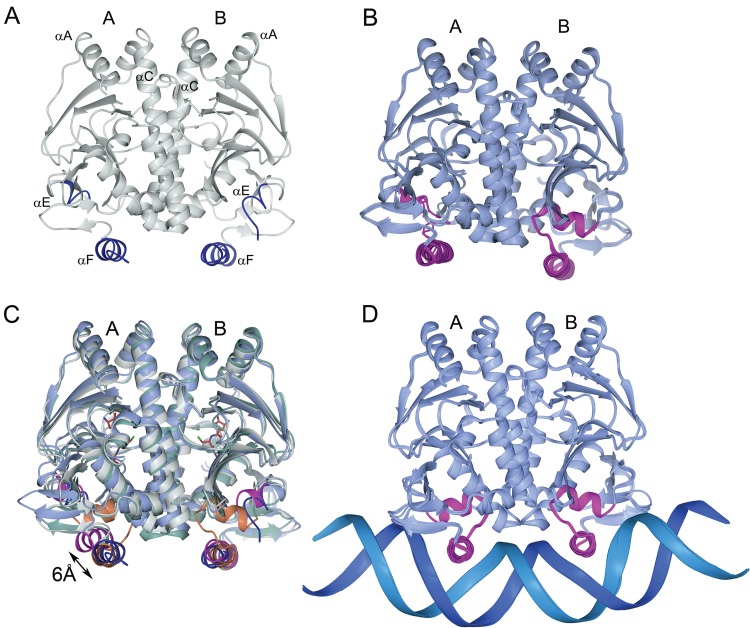
Superimposed structures of PrfA. All superimpositions are based on residues 2 to 237 in monomer B. For root-mean-square (rms) deviations, see Table S2. (A) The PrfA_WT_ homodimer (PDB code 2BEO [17]). The HTH motifs are shown in dark blue; only the recognition helix (αF) is folded. (B) Superimposed structures of the four intermediately activated structures of PrfA studies: PrfA_A94V_, PrfA_L140H_, PrfA_L140F_, and PrfA_A218G_. Only the HTH motifs in monomer B are folded. (C) Superimposed structures of PrfA_WT_, PrfA_G145S_, and one representative of the intermediately activated fold (PrfA_A218G_). Noteworthy is the shift with up to 6 Å in the position of the recognition helix in monomer A in PrfA_A218G_ compared to that of monomer A in PrfA_G145S_. The C-alpha trace of PrfA_G145S_ is shown in green, with the folded HTH motifs shown in orange. (D) Seven structures of PrfA in complex with DNA: PrfA_WT_ (PDB code 5LEJ [10]), PrfA_WT_-GSH (PDB code 5LRS [10]), PrfA_G145S_ (PDB code 5LEK [10]), PrfA_A94V_, PrfA_L140H_, PrfA_L140F_, and PrfA_A218G_. Note the close to identical structures of all PrfAs when in complex with DNA (rms deviations < 0.5 Å).

PrfA_WT_ is a homodimer in which each monomer consists of an N-terminal domain (residues 1 to 108) and a C-terminal DNA-binding domain (residues 138 to 237) linked by a long α-helix (αC, residues 109 to 137) (17). Both the N- and C-terminal domains constitute an α/β-fold. Hydrophobic interactions between symmetry-related αC helices and loops β6-β7 stabilize the dimer interface. Two α-helices in the C-terminal domain, αE (residues 170 to 178) and αF (residues 183 to 197), constitute the two helices of the typical HTH motif present in many prokaryotic transcription factors. In PrfA_WT_, parts of the first helix and the connecting turn of the HTH motif were not defined by electron density, probably due to high flexibility (17) (Fig. 5A). A comparison of the structure of the constitutively active mutant PrfA_G145S_ with that of PrfA_WT_ revealed the first details of the structural differences between the inactive and active forms of PrfA (17). These changes have also been verified in the activated PrfA_WT_-GSH complex structure (PDB code 5LRR [10]). In activated PrfA, the HTH motif is folded; however, activation also leads to a more collapsed structure (17).

We expected that the crystal structures of PrfA_L140H_, PrfA_L140F_, and PrfA_A218G_ would be similar to the PrfA_G145S_ structure, since our *in vivo* characterization classified them as PrfA* mutations. However, their structures showed that each of the PrfA* variants had only one folded HTH motif, with the remaining structure residing in the PrfA_WT_ conformation (Fig. 5B). Hence, the new mutant structures display features characteristic of both the inactive and active forms of PrfA. It is noteworthy that we did not manage to get crystals of the PrfA_WT_ under these crystallization conditions; however, crystals of PrfA_A94V_, which showed almost PrfA_WT_ activity levels (Fig. 2), were obtained. The PrfA_A94V_ protein also had one folded HTH and one unfolded (WT) motif identical to that of the PrfA* mutants (Fig. 5B). Thus, the new structures of PrfA* variants and the wild-type-like PrfA_A94V_ protein, presented here, represent an intermediately activated form of the protein. The most striking structural difference between the inactive, intermediate-active, and fully active structures is the position of their recognition helices. When superimposed on monomer B, the recognition helix in monomer A is shifted up to 6 Å (Fig. 5C). However, when PrfA is bound to promoter DNA, both monomers are almost identical to each other in all of our PrfA mutants (Fig. 5D).

## DISCUSSION

The rationale for this work was to further our understanding of the mechanism by which PTS sugars repress PrfA activity (1, 11–13). We therefore undertook a transposon-based strategy to isolate mutants with a PrfA-activated phenotype. To do this, we selected for growth of L. monocytogenes in medium containing glucose-6-phosphate as the only carbon source. Most transposon mutants that were able to grow under these conditions also carried base substitution mutations in the *prfA* gene, resulting in amino acid substitutions and constitutively activated forms of PrfA (PrfA*). Interestingly, they showed a varying degree of PrfA activation, as judged by “classical” PrfA* phenotypes such as fosfomycin sensitivity, virulence factor expression, bacterial uptake into eukaryotic cells, and intracellular growth. Also, the PrfA* proteins that we isolated also showed a diverse ability to bind the promoter regions of different PrfA-regulated genes. Conclusively, our isolated PrfA* mutants ressemble phenotypes of other previously characterized PrfA* mutants (15).

We determined the crystal structures of the medium- to highly activated PrfA* mutants to see if their various activation levels were reflected structurally. The PrfA_WT_ strain has flexible HTH domains allowing for only weak DNA binding, whereas the fully activated PrfA*_G145S_ mutant and the glutathione-activated PrfA_WT_ strain have structured HTH domains that allow maximal DNA binding (10, 17). In addition to folded HTH motifs, the activated PrfA proteins also have a collapsed central core structure (17). We found that the structures of the PrfA* proteins in this study had mixtures of structural features characteristic of both inactive and active PrfA. The PrfA_L140H_, PrfA_L140F_, and PrfA_A218G_ PrfA* mutants all had a folded HTH motif in one monomer of the homodimer but an uncollapsed central core structure. Consequently, we suggest that these mutant structures represent an intermediately activated form of the protein with features characteristic of both inactive and activated PrfA. We hypothesize that this form is also present during activation of PrfA_WT_ or PrfA_G145S_ but too short-lived to be captured in crystal structures. Taken together, these experiments give a structural explanation of why there exist different activity levels of the PrfA* mutants.

By adopting an ensemble of interconverting conformational states under physiological conditions, transcriptional activators like PrfA are primed to quickly switch their function in response to changes in their environments.

## MATERIALS AND METHODS

### Bacterial strains and plasmids.

The bacterial strains and plasmids are listed in Tables S3 and S4 in the supplemental material. Listeria monocytogenes EGDe (serotype 1/2a) strains were subcultured in BHI (Thermo Fisher Scientific), in BHI supplemented with 1% (wt/vol) Amberlite XAD4 (06444; Fluka) (BHIA) (24), or in a chemically defined medium (DM) as described by Amezaga et al. (26). Escherichia coli strains were subcultured in Luria-Bertani (LB) medium. Antibiotics were added as appropriate.

### Genetic manipulation.

All oligonucleotides are listed in Table S5. The pMAD vector (Eurofins) was used to delete the *hpt* allele as described previously (27). Construction of the G145C, L140F, L140H, A94V, and A218G *prfA* mutants was performed as follows: 10 ng of plasmid pLis35 (28) or pET-His1a-*prfA* was amplified using either primer pair PrfAG145Cfwd and PrfAG145Crev (for G145C), PrfAL140Ffwd and PrfAL140Frev (for L140F), PrfAL140Hfwd and PrfAL140Hrev (for L140H), PrfAA94Vfwd and PrfAA94Vrev (for A94V), or PrfAA218Gfwd and PrfAA218Grev (for A218G). Afterward, the PCR products were digested using 10 U of DnpI (Thermo Scientific) at 37°C for 1 h. The reaction mixture was transformed into E. coli strain DH5α. The point mutations were verified by sequencing the *prfA* gene.

### Glucose-6-phosphate screen.

A previously constructed *Himar1-mariner* transposon (Tn) mutant library (20) was inoculated into 96-well plates containing BHI and subsequently grown at 37°C with shaking overnight (o/n). The following day, the cultures were spun down and washed three times in 1× PBS before they were replica plated into 96-well plates containing DM supplemented with 0.2% glucose-6-phosphate (G-6-P). The bacteria were grown at 37°C with shaking for 2 days. The cultures were examined daily for growth, and the Tn mutants that showed growth were recovered by restreaking onto BHI plates. To verify their ability to grow in the screening conditions, the colonies were reinoculated into DM supplemented with 0.2% G-6-P in 15-ml Falcon tubes and grown at 37°C with shaking. In addition, the colonies were streaked onto blood agar plates to examine their hemolytic ability.

### Fosfomycin sensitivity test.

Fosfomycin sensitivity was determined by using 100 μg fosfomycin antibiotic discs (Liofilchem). BHI plates with or without 7 μg/ml chloramphenicol were inoculated using swabs soaked with a bacterial suspension in sterile saline. The suspension was adjusted to a turbidimetry of an optical density at 600 nm (OD_600_) of 0.5 on the MacFarland scale. A fosfomycin antibiotic disc was placed on the plates, and they were incubated overnight at 37°C. The next day, the inhibition zone was measured.

### Intracellular growth.

Human colon epithelial Caco-2 cells were seeded into 24-well dishes (Corning BioCoat Cellware; collagen type I, VWR International) at a density of 8 · 10^4^ cells per well and infected o/n with cultures of bacterial strains at a multiplicity of infection of 10. The cells and bacteria were centrifuged at 130 × *g* for 5 min to synchronize the infection. At 1 h postinfection, the Caco-2 cells were washed twice with PBS. Cell growth medium supplemented with 50 μg/ml gentamicin was added to kill extracellular bacteria. At 2, 4, and 6 h postinoculation, the cells were lysed in water, and the bacteria were plated on Luria agar (LA) plates.

### Plaque assay.

A plaque assay was performed as described previously, with some modifications (29) giving a more qualitative number of infectivity. Briefly, TC7 cells (a kind gift from Andrea Puhar, Umeå University, Sweden) were seeded into six-well dishes (Corning BioCoat Cellware; collagen type I, VWR) at a density of 10^6^ cells/well and were infected with a multiplicity of infection of 1:500 with PBS-washed overnight cultures grown in BHI at 37°C with shaking. Two hours postinfection, the TC7 monolayer was washed, and an agarose overlay was added, consisting of Dulbecco modified Eagle medium (DMEM), 0.7% agarose, 20% fetal calf serum, and 50 μg/ml gentamicin. Two days postinfection, the agarose overlay was removed, and the cells were fixed with absolute ethanol for 5 min and stained with Giemsa before the plaques were counted.

### Isolation of RNA.

Isolation of RNA was performed essentially as previously described in reference 30. Overnight cultures were diluted 50-fold in BHI and incubated at 37°C with shaking and grown until they reached an OD_600_ of 1.0. The bacteria were collected by centrifugation at 4°C and 6,000 × *g* for 10 min and frozen at –80°C. Pelleted bacteria were resuspended in resuspension solution (10% glucose, 12.5 mM Tris-HCl [pH 7.6], 5 mM EDTA). After transfer of the samples to a bead beater tube containing 0.4 g glass beads and 0.5 ml phenol (pH 4.5), the cells were homogenized in a mini-bead beater (BioSpect Products) for 75 s. The mix was then centrifuged for 5 min at 16,800 × *g* at 4°C before the addition of 1 ml TRIzol (Ambion) and 100 μl of a 24:1 ratio of chloroform-isoamyl alcohol added to the aqueous phase. The samples were centrifuged for 5 min at 16,800 × *g* at 4°C. After centrifugation, two more chloroform-isoamyl alcohol extractions were performed before precipitation of the RNA by the addition of 0.7 volume of isopropanol, and it was placed in the freezer for 30 min. The sample was then centrifuged for 20 min at 4°C and 16,800 × *g*. The dried RNA pellet was dissolved in 200 μl diethyl pyrocarbonate (DEPC)-treated water. Samples were subjected to DNase I treatment (20 U) and incubated for 30 min at 37°C. The reaction was stopped by addition of phenol-chloroform-isoamyl alcohol (Ambion) and centrifuged for 5 min at 16,800 × *g* and 4°C. The aqueous phase was extracted with chloroform-isoamyl alcohol as described above before centrifugation. The purified RNA was pelleted with the addition of 1/10 volume of DEPC-treated 3M NaOH (pH 4.5) and 2.5 volumes of 99.5% ethanol, incubated at –20°C for 30 min, and pelleted by centrifugation at 16,800 × *g* (4°C for 20 min). The RNA was dissolved in 200 μl of DEPC-treated water. The extracted RNA was analyzed on a 1.2% agarose gel to verify transcript integrity. The concentration of the RNA was measured on a NanoDrop 1000 spectrophotometer.

### Northern blots.

Northern blotting was performed as described previously (19). In brief, 25 μg of RNA was separated on an agarose gel (1.2% agarose, 1× HEPES buffer [20 mM HEPES, 5 mM sodium acetate, 1 mM EDTA, adjusted to pH 7], 7.3% formaldehyde). The gel was run in 1× HEPES buffer at 100 V for 4 h, and the RNA was transferred to a Hybond-N membrane (GE Healthcare) by capillary transfer in 20× SSC buffer (1× SSC is 0.15 M NaCl plus 0.015 M sodium citrate). The membranes were cross-linked using UV light, prehybridized at 50°C (for *hpt*) or 60°C (for transfer messenger RNA [tmRNA]) in Rapid hyb buffer (GE Healthcare) for about 2 h, and then hybridized with DNA probes at 50°C or 60°C overnight, respectively. Membranes were washed (0.1% sodium dodecyl sulfate, 2× SSC) at room temperature for 15 min, followed by a second wash (0.1% SDS, 0.1× SSC) at 50°C or 60°C for 15 min. Thereafter, the membranes were exposed in a phosphorimager cassette and developed using the Typhoon FLA9500 scanner (GE Healthcare). The probes were created by amplifying genomic L. monocytogenes EGDe DNA with PCR and primers uhpT-U/uhpT-D for *uhpT* and tmRNA-U/tmRNA-D for tmRNA. The primer sequences are in shown in Table S5. Probes were subsequently labeled with α-P^32^ dATP (PerkinElmer) using the Megaprime DNA labeling system (GE Healthcare) according to the manufacturer’s instructions.

### Western blots.

Western blots were performed as described previously, with minor changes (19). Bacterial cultures were grown in BHI supplemented with the appropriate antibiotics at 37°C with shaking. At an OD_600_ of 1.0, the cultures were processed either as a whole-cell fraction or as a secreted fraction as follows.

**Supernatant fraction.** First, 1 ml of the culture supernatant was precipitated trichloroacetic acid; a one-fourth volume of ice-cold 50% trichloroacetic acid was added to the samples, which were then incubated on ice for 1 h. The samples were spun down (10 min, 16,800 × *g*), and the precipitate was washed in 80% ice-cold acetone. The dried protein pellets were suspended in 1× Laemmli buffer (31) and run on SDS-PAGE and Western blotting. The Western blot was developed with rabbit anti-LLO (ab43018; Abcam), horseradish peroxidase (HRP)-conjugated goat anti-rabbit secondary antibodies (as09602; Agrisera), and HRP-conjugated rabbit anti-mouse secondary antibodies (Dako P0260).

**Whole-cell fraction.** The cultures were added to an equal volume of 1:1 ethanol-acetate and frozen at –20°C o/n. Subsequently, the samples were centrifuged, and the bacterial pellet was lysed in lysis buffer (20 mM Tris-HCl, pH 8.0; 50 mM EDTA, pH 8.0; 20% sucrose) with added lysozyme and DNase. The samples were heated at 37°C for 1 h and run on SDS-PAGE and Western blotting. The Western blot was developed using anti-ActA (19), anti-PrfA R79IS4b (kindly provided by Pascale Cossart, Institute Pasteur, Paris, France), anti-RNA polymerase beta (RpoB) (ab202891; Abcam), and HRP-conjugated secondary antibodies (as09602; Agrisera) or anti-RpoB (BioSite) and HRP-conjugated rabbit anti-mouse secondary antibodies (Dako P0260).

### Amberlite induction.

Overnight L. monocytogenes cultures grown in BHI supplemented with the appropriate antibiotic were diluted into BHI supplemented with 1% (wt/vol) Amberlite XAD4 (06444; Fluka) and grown at 37°C until an OD_600_ of 1.0 was achieved. Samples were processed as described under “Western blots.”

### Surface plasmon resonance (SPR).

The interaction study was performed using a ProteOn XPR36 biosensor (Bio-Rad, USA) equipped with an NLC sensor chip (Bio-Rad, USA). Biotinylated double-stranded DNA (dsDNA) strands 5′-TTTTGTTTTCTGCATGATAACAAGTGTTAATGACGGAAAG-3′ (*hpt* promoter), 5′-AGTTGGGGTTAACTGATTAACAAATGTTAGAGAAAAATTA-3′ (*act* promoter), and 5′-CTTTTATGTTGAGGCATTAACATTTGTTAACGACGATAAA-3′ (*hly* promoter) were immobilized to a density of 50 to 90 response units (RU). All SPR experiments were performed at 25°C in 30 mM Tris-HCl (pH 7.4) containing 200 mM NaCl and 0.05% Tween 20. A blank surface or interspot region was used as a reference and subtracted from the data. Graded concentrations of PrfA and its derivatives were injected over the different promoters. The rate and dissociation constants were derived by global fitting of at least four different PrfA concentrations with ProteOn software (Bio-Rad, USA).

### Protein expression and purification.

The PrfA_L140F_, PrfA_L140H_, PrfA_A94V_, and PrfA_A218G_ constructs, cloned as described above, encode the full-length PrfA protein (M1-N237) as well as a 6-His tag and a tobacco etch virus (TEV) protease cleavage site. This results in the addition of two nonnative N-terminal residues (GA) upon TEV cleavage. Proteins were overexpressed in E. coli BL21(DE3)plysS cells (Novagen) grown at 37°C in LB medium, supplemented with 50 μg/ml kanamycin and 34 μg/ml chloramphenicol, and then induced with isopropyl-β-d-1-thiogalactopyranoside (final concentration of 0.4 mM) at an OD_600_ of 0.6. Growth was continued o/n at 20°C, and cells were then harvested by centrifugation and lysed by sonication on ice.

Purification of PrfA proteins for assays and crystallization was performed using Ni-NTA Superflow FF (Qiagen) in a lysis buffer containing 50 mM sodium phosphate (pH 8.0), 20 mM imidazole, and 500 mM NaCl. The columns were washed with 10 column volumes of lysis buffer followed by 10 column volumes of 50 mM sodium phosphate (pH 8.0) and 1,000 mM NaCl before elution of PrfA proteins with 50 mM sodium phosphate (pH 8.0), 300 mM imidazole, and 500 mM NaCl. The polyhistidine tag was removed by overnight cleavage with TEV protease at 4°C in 50 mM sodium phosphate (pH 7.1) and 200 mM NaCl. Cleaved target proteins were separated from the 6-His-tagged TEV protease, 6-His tag fragments, and uncleaved target proteins by nickel affinity chromatography as described above. The eluted target protein was dialyzed into a final buffer consisting of 20 mM Tris-HCl (pH 7.1) and 100 mM NaCl.

### Crystallization of PrfA* mutants and the mutants in complexes with the *hly* promoter DNA.

For crystallization screening, proteins were additionally purified by ion exchange (GE Healthcare) and size exclusion chromatography. Prior to ion exchange, the sample pH was adjusted to 6.5, and the MonoS 5/5 column (GE Healthcare) was eluted with a linear gradient of 200 to 650 mM NaCl in 20 mM sodium phosphate (pH 6.5). Purified proteins were eluted at ∼250 mM NaCl. The peak fractions of PrfA were pooled and applied to a HiLoad Superdex 75 16/60 column (GE Healthcare) equilibrated with 50 mM sodium phosphate (pH 6.5) and 200 mM NaCl. Proteins used for cocrystallization with *hly* DNA were further buffer-exchanged to 20 mM Tris-HCl (pH 8.0) and 150 mM NaCl. Each protein was concentrated using Amicon Ultra centrifugal filter devices (Millipore) before being flash-frozen in liquid N_2_ and stored at –80°C.

Two complementary 30-bp DNA oligonucleotides, representing the *hly* PrfA box motif, obtained from Eurofins Genomics (5′-TTGAGGCATTAACATTTGTTAACGACGATA-3′, reverse complement: 5′-TATCGTCGTTAACAAATGTTAATGCCTCAA-3′) were annealed by cooling from 95°C to room temperature over 3 h in 10 mM Tris-HCl (pH 8.0), 50 mM NaCl, and 1 mM EDTA. This formed a blunt-ended DNA duplex.

The PrfA*variants were crystallized by the hanging-drop vapor-diffusion method in VDX plates (Hampton Research) at 18°C. Droplets of 2 to 4 μl protein solution at 3 mg/ml were mixed with 2 μl reservoir solution consisting of 24% polyethylene glycol 4000, 100 mM sodium citrate (pH 5.5), and 17% isopropanol. Crystals used for data collection were obtained after 2 to 5 days. For crystallization of PrfA-DNA complexes, the protein and *hly* PrfA box motif duplex DNA were incubated together at a ratio of 1:1.3 (PrfA dimer-*hly* DNA) at final concentrations of 50 μM and 70 μM, respectively, in 20 mM Tris-HCl (pH 8.0) and 150 mM NaCl for 30 min at room temperature before crystallization screening. Crystals were obtained after 24 h by mixing 4 μl protein-DNA solution with 2 μl reservoir solution consisting of 8% polyethylene glycol 8000, 100 mM sodium acetate (pH 4.6), 100 mM magnesium acetate, and 20% glycerol. Crystals of PrfA-DNA complexes were cryoprotected in reservoir solution supplemented with 30% glycerol before vitrification in liquid nitrogen. PrfA crystals were vitrified directly from their drop solutions.

### Data collection and structure determinations.

Diffraction data were collected at –173°C at the European Synchrotron Radiation Facility (ESRF) beamline ID30-B. Images were processed with X-ray detector software (XDS) (32, 33) and subsequently scaled and merged using Aimless, a component of the CCP4 software suite (34). Structures were solved by molecular replacement using the previously determined structures of PrfA_WT_ (PDB code 5F1R [19]), PrfA_G145S_ (PDB code 2BGC [17]), and the PrfA_WT_-hly DNA complex (PDB code 5LEJ [10]) as search models with the program Phaser from the PHENIX suite (35). Atomic models were iteratively rebuilt manually and refined using the programs Coot (36) and phenix.refine (35). Bases of the two chains of the palindromic *hly* PrfA box motif DNA are numbered from –15 to +15. Data collection, refinement, and validation statistics are presented in Table S1. Superimpositions are based on all main chain atoms of residues 2 to 237 using the program SSM (37). All structural figures were prepared with CCP4mg (38).

### Data availability.

The atomic coordinates and the structure factors have been deposited in the Protein Data Bank under the PDB codes 6QVY (for PrfA_A94V_), 6QVZ (for PrfA_L140H_), 6QW1 (for PrfA_L140F_), 6QW2 (for PrfA_A218G_), 6QWF (for PrfA_A94V_-DNA), 6QWH (for PrfA_L140H_-DNA), 6QWK (for PrfA_L140F_-DNA), and 6QWM (for PrfA_A218G_-DNA).

## Supplementary Material

Supplemental file 1
