# Supplementary material for: A Novel Growth-Based Selection Strategy Identifies New Constitutively Active Variants of the Major Virulence Regulator PrfA in Listeria monocytogenes
Source: J Bacteriol. 2020 May 11;202(11):e00115-20. doi: 10.1128/JB.00115-20 (PMC7221254; doi:10.1128/JB.00115-20)
Supplement: Supplemental file 1 [file JB.00115-20-s0001.pdf]

A novel growth based selection strategy identifies new constitutively active variants of the major virulence regulator PrfA in *Listeria monocytogenes*

Sabine Hansen[a,c], Michael Hall[b,c], Christin Grundström[b,c], Kristoffer Brännström[d], A. Elisabeth Sauer-Eriksson[b,c], Jörgen Johansson[a,c,e]

[a]*Department of Molecular Biology;* [b]*Department of Chemistry;* [c]*Umeå Centre for Microbial research (UCMR);* [d]*Department of Medical Biochemistry and Biophysics;* [e]*Molecular Infection Medicine, Sweden (MIMS), Umeå University, 901 87 Umeå, Sweden*

Address correspondence to sabine.hansen@umu.se, elisabeth.sauer-eriksson@umu.se, or jorgen.johansson@umu.se

J.J. ORCID: http://orcid.org/0000-0002-0904-497X

E. S.-E. ORCID: http://orcid.org/0000.0003.0124.0199

Running title: PrfA can exist in an intermediate-activated form.

TABLE S1 Data collection and refinement statistics[a]

	$PrfA_{A94V}$	$PrfA_{L140H}$	$PrfA_{L140F}$	$PrfA_{A218G}$
Space group	$P2_12_12_1$	$P2_12_12_1$	$P2_12_12_1$	$P2_12_12_1$
Unit-cell parameters (Å)	a=48.5, b=88.6, c=116.0	a=47.8, b=88.5, c=116.1	a=47.6, b=88.8, c= 115.4	a=47.8, b=88.0, c= 116.4
Resolution limits (Å)	48.5-1.69 (1.75-1.69)	48.5-2.7 (2.8-2.7)	48.4-1.84 (1.88-1.84)	44.0-2.6 (2.7-2.6)
No. of unique reflections	55739 (5391)	13826 (1733)	42882 (2364)	15707 (1540)
Multiplicity	13.4 (12.8)	6.2 (6.1)	13.4 (13.1)	13.4 (13.9)
Completeness (%)	99.5 (94.0)	98.5 (94.7)	99.5 (91.6)	99.9 (99.9)
R_{merge}	0.098 (1.76)	0.146 (0.715)	0.082 (1.417)	0.231 (1.86)
R_{pim}	0.040 (0.72)	0.094 (0.460)	0.033 (0.573)	0.093 (0.742)
$\langle I/\sigma(I)\rangle$	14.9 (1.5)	8.4 (2.0)	17.7 (1.9)	10.3 (1.8)
**Refinement and model building statistics**				
R factor (%)	15.0 (23.0)	18.94 (26.87)	18.30 (28.76)	18.49 (27.11)
R free (%)	20.9 (34.4)	24.57 (30.83)	24.19 (35.24)	25.25 (42.89)
No. of protein atoms	3938	3821	3898	3820
No. of water molecules	315	142	318	203
No. of atoms: 2-propanol/ glycerol/Na^+	24/0/3	16/0/3	28/0/3	16/0/2
**Rms deviations from ideal geometry**				
Bond length (Å)	0.011	0.003	0.012	0.005
Bond angles (°)	0.99	0.51	1.14	0.50
Ramachandran plot:				
Residues in most favored regions (%)	98.0	98.0	98.0	97.0
Residues in disallowed regions (%)	0.0	0.0	0.0	0.0
Clashscore	2.4	2.6	2.0	0.9
Average B-factor ($Å^2$):				
Protein	35.9	47.7	44.8	49.1
Ligands	41.1	41.1	47.1	47.9
Water	42.4	42.5	47.6	44.1
PDB ID	6QVY	6QVZ	6QW1	6QW2

[a] Values in parenthesis are for the highest resolution shell

TABLE S1 Data collection and refinement statistics, continue[a]

	$PrfA_{A94V}$-DNA	$PrfA_{L140H}$-DNA	$PrfA_{L140F}$-DNA	$PrfA_{A218G}$-DNA
Space group	$P4_32_12_1$	$P4_32_12_1$	$P4_32_12_1$	$P4_32_12_1$
Unit-cell parameters (Å)	a=78.4, b=78.4, c= 265.4	a=78.3, b=78.3, c= 265.0	a=78.4, b=78.4, c= 265.3	a=78.6, b=78.6, c= 265.1
Resolution limits (Å)	39.2-2.7 (2.80-2.70)	39.1-2.9 (3.00-2.90)	42.5-1.9 (3.00-2.90)	47.1-2.9 (3.00-2.90)
No. of unique reflections	23581 (2304)	18986 (1859)	19158 (1877)	19313 (1891)
Multiplicity	26.8 (28.3)	25.6 (28.8)	25.6 (28.0)	25.7 (27.8)
Completeness (%)	99.9 (100.0)	99.1 (99.8)	99.5 (99.8)	99.8 (99.9)
R_{merge}	0.097 (1.367)	0.125 (2.188)	0.175 (2.059)	0.137 (0.976)
R_{pim}	0.026 (0.363)	0.035 (0.579)	0.048 (0.550)	0.038 (0.261)
<I/σ (I)>	21.2 (3.2)	21.5 (2.1)	14.6 (2.2)	18.2 (4.0)
**Refinement and model building statistics**				
R factor (%)	22.5 (30.8)	25.4 (34.7)	23.3 (34.6)	23.6(34.2)
R free (%)	26.4 (38.5)	28.9 (43.1)	28.2 (43.3)	29.0(43.6)
No. of protein/DNA atoms	5085	5074	5087	5079
No. of water molecules	24	22	22	11
No. of atoms: 2-propanol/Na^+/glycerol	0/0/12	0/0/6	0/0/6	0/0/18
**Rms deviations from ideal geometry**				
Bond length (Å)	0.003	0.004	0.003	0.004
Bond angles (°)	0.51	0.58	0.55	0.58
Ramachandran plot:				
Residues in most favored regions (%)	96.0	94.0	94.0	94
Residues in disallowed regions (%)	0.0	0.0	0.0	0.0
Clashscore	1.9	2.6	1.6	1.1
Average B-factor ($Å^2$):				
Protein	75.8	98.6	87.2	74.4
Ligands	84.8	102.3	95.2	82.6
Water	64.2	76.3	57.9	47.3
PDB ID	6QWF	6QWH	6QWK	6QWM

[a] Values in parenthesis are for the highest resolution shell

TABLE S2 Superimposition of PrfA structures[a]. Root-mean-square (rms) deviations vary between 0.8-1.2 Å superimposing residues 2-237 of the PrfA* mutations characterized in this study with the same residues in $PrfA_{WT}$, and between 1.4-2.0 Å superimposing the PrfA* mutants with $PrfA_{G145S}$ or $PrfA_{WT}$-GSH. Rms deviations lower than 1.3 Å are highlighted in bold.

**PDB ID**<br>**Mutants**<br>**Monomers**	**2BEO**<br>**$PrfA_{WT}$**<br>**A (B)**	**5F1R[b]**<br>**$PrfA_{WT}$-C10**<br>**A (B)**	**2BGC**<br>**$PrfA_{G145S}$**<br>**A (B)**	**5LRR**<br>**$PrfA_{WT}$-GSH**<br>**A (B)**	**5LEJ**<br>**$PrfA_{WT}$-DNA**<br>**A (B)**
$PrfA_{A94V}$ (A)	**1.16 (1.20)**	**0.95 (1.30)**	1.78 (2.04)	1.67 (1.77)	1.82 (1.81)
$PrfA_{A94V}$ (B)	**0.88 (0.89)**	**1.02 (0.99)**	1.43 (1.53)	1.38 (1.37)	1.45 (1.44)
$PrfA_{L140H}$ (A)	**1.18 (1.28)**	**0.96 (1.30)**	1.79 (1.96)	1.73 (1.79)	1.83 (1.82)
$PrfA_{L140H}$ (B)	**0.84 (0.86)**	**0.96 (1.15)**	1.46 (1.55)	1.42 (1.41)	1.51 (1.46)
$PrfA_{L140F}$ (A)	**1.22 (1.24)**	**0.99 (1.29)**	1.82 (2.03)	1.73 (1.77)	1.82 (1.81)
$PrfA_{L140F}$ (B)	**0.85 (0.89)**	**1.01 (1.00)**	1.46 (1.58)	1.42 (1.48)	1.50 (1.54)
$PrfA_{A218G}$ (A)	**1.13 (1.23)**	**0.90 (1.30)**	1.75 (1.94)	1.71 (1.78)	1.81 (1.79)
$PrfA_{A218G}$ (B)	**0.89 (0.83)**	**0.76 (0.95)**	1.52 (1.65)	1.47 (1.52)	1.57 (1.60)
$PrfA_{A94V}$-DNA (A)	1.50 (1.58)	1.56 (1.68)	**0.66 (0.61)**	**0.61 (0.43)**	**0.32 (0.35)**
$PrfA_{A94V}$-DNA (B)	1.51 (1.61)	1.56 (1.94)	**0.67 (0.61)**	**0.63 (0.46)**	**0.37 (0.26)**
$PrfA_{L140H}$-DNA (A)	1.56 (1.56)	1.54 (1.62)	**0.70 (0.71)**	**0.63 (0.47)**	**0.45 (0.47)**
$PrfA_{L140H}$-DNA (B)	1.42 (1.50)	1.48 (1.66)	**0.72 (0.76)**	**0.66 (0.53)**	**0.47 (0.48)**
$PrfA_{L140F}$-DNA (A)	1.47 (1.50)	1.55 (1.60)	**0.68 (0.61)**	**0.65 (0.47)**	**0.35 (0.39)**
$PrfA_{L140F}$-DNA (B)	1.52 (1.61)	1.61 (1.95)	**0.67 (0.60)**	**0.67 (0.53)**	**0.65 (0.30)**
$PrfA_{A218G}$-DNA (A)	1.45 (1.54)	1.50 (1.58)	**0.66 (0.71)**	**0.62 (0.48)**	**0.44 (0.48)**
$PrfA_{A218G}$-DNA (B)	1.48 (1.59)	1.54 (2.06)	**0.65 (0.65)**	**0.63 (0.50)**	**0.64 (0.38)**

**PDB ID**<br>**Mutants**<br>**Monomers**	**2BEO**<br>**$PrfA_{WT}$**<br>**A (B)**	**5F1R[b]**<br>**$PrfA_{WT}$-C10**<br>**A (B)**	**2BGC**<br>**$PrfA_{G145S}$**<br>**A (B)**	**5LRR**<br>**$PrfA_{WT}$-GSH**<br>**A (B)**	**5LEJ**<br>**$PrfA_{WT}$-DNA**<br>**A (B)**
2BEO (A)	-	**0.55 (0.69)**	1.55 (1.57)	1.43 (1.45)	1.50 (1.50)
2BEO (B)	-	**0.69 (0.54)**	1.55 (1.64)	1.49 (1.53)	1.57 (1.63)
5F1R (C10) (A)		-	1.50 (1.67)	1.37 (1.50)	1.62 (1.58)
5F1R (C10) (B)		-	1.72 (1.76)	1.50 (1.63)	1.58 (1.98)
2BGC (A)			-	**0.52 (0.60)**	**0.65 (0.67)**
2BGC (B)			-	**0.77 (0.62)**	**0.56 (0.58)**
5LRR (A)				-	**0.65 (0.69)**
5LRR (B)				-	**0.50 (0.51)**

[a] Superimpositions (SSM) are based on the main chain atoms of residues 2-237. Rms deviations less than 1.3 Å are highlighted in bold.

[b] This structure is $PrfA_{WT}$ in complex with a ring-fused 2-pyridone inhibitor (1) and represent here a second independent structure of the $PrfA_{WT}$ conformation.

TABLE S3 Bacterial strains used in this study

Strain	Description	Ref
EGDe	*Listeria monocytogenes*	
EGDe G145S$_{native}$	EGDe PrfAG145S	(2)
EGDe G145S$_{native}$ Δ*uhpT*	EGDe PrfAG145S Δ*uhpT*	This study
WT	EGDe Δ*prfA* pPL2*prfA*	This study
G145S	EGDe Δ*prfA* pPL2*prfAG145S*	This study
G145C	EGDe Δ*prfA* pPL2*prfAG145C*	This study
L140F	EGDe Δ*prfA* pPL2*prfAL140F*	This study
L140H	EGDe Δ*prfA* pPL2*prfAL140H*	This study
A94V	EGDe Δ*prfA* pPL2*prfAA94V*	This study
A218G	EGDe Δ*prfA* pPL2*prfAA218G*	This study
ΔactA	EGDe Δ*actA* pPL2	This study
Δhly	EGDe Δ*hly* pPL2	This study
ΔprfA	EGDe Δ*prfA* pPL2	This study
Tn	Mariner-based transposon library	(3)

TABLE S4 Plasmids used in this study

Plasmids	Description	Ref
pMADdeltauhpT	*uhpT* deletion plasmid	Eurofins
pPL2	Expression vector	(4)
pLis35	pPL2 carrying *prfA*$_{WT}$	(5)
pKVA609	pPL2 carrying *prfA*$_{G145S}$	(2)
pPL2prfAG145C	pPL2 carrying *prfA*$_{G145C}$	This study
pPL2prfAL140F	pPL2 carrying *prfA*$_{L140F}$	This study
pPL2prfAL140H	pPL2 carrying *prfA*$_{L140H}$	This study
pPL2prfAA94V	pPL2 carrying *prfA*$_{A94V}$	This study
pPL2prfAA218G	pPL2 carrying *prfA*$_{A218G}$	This study
pET His1a PrfA	PrfA$_{WT}$ overexpression vector	(1)
pET His1a PrfAG145S	PrfA$_{G145S}$ overexpression vector	This study
pET His1a PrfAL140F	PrfA$_{L140F}$ overexpression vector	This study
pET His1a PrfAL140H	PrfA$_{L140H}$ overexpression vector	This study
pET His1a PrfAA94V	PrfA$_{A94V}$ overexpression vector	This study
pET His1a PrfAA218G	PrfA$_{A218G}$ overexpression vector	This study

TABLE S5 Oligonucleotides used in this study

Oligonucleotide name	Sequence	Characteristics
**Oligonucleotides used for Surface Plasmon Resonance**		
hly_{fw}	CTTTTATGTTGAGGCATTAACATTTGTTAACGACGATAAA	5'-Biotinylated
hly_{rev}	TTTATCGTCGTTAACAAATGTTAATGCCTCAACATAAAAG	
hpt_{fw}	TTTTGTTTTCTGCATGATAACAAGTGTTAATGACGGAAAG	5'-Biotinylated
hpt_{rev}	CTTTCCGTCATTAACACTTGTTATCATGCAGAAAACAAAA	
$actA_{fw}$	AGTTGGGGTTAACTGATTAACAAATGTTAGAGAAAAATTA	5'-Biotinylated
$actA_{rev}$	TAATTTTTCTCTAACATTTGTTAATCAGTTAACCCCAACT	
**Oligonucleotides used to create base-substitution mutations**		
PrfA218Gfwd2	5´-TCTCAAAAGATATGGCCCTAAATTAGATG-3´	5´ A218G Quickchange
PrfA218Grev2	5´-CATCTAATTTAGGGCCATATCTTTTGAGA-3´	3´ A218G Quickchange
PrfAA94Vfwd	5´-CATTAGCGAGCAGGCTACCGTATACGTTATCAAAATAAACGAACTAAAAG-3´	5´ A94V Quickchange
PrfAA94VREV	5´-CTTTTAGTTCGTTTATTTTGATAACGTATACGGTAGCCTGCTCGCTAATG-3´	3´ A94V Quickchange
PrfAL140Ffwd	5´-GATTTTTCGATTAACGGGAAGTTTGGCTCTATTTGCGGTCAAC-3´	5´ L140F Quickchange
PrfAL140Frev	5´-GTTGACCGCAAATAGAGCCAAACTTCCCGTTAATCGAAAAATC-3´	3´ L140F Quickchange
PrfAL140Hfwd	5´-CGATTAACGGGAAGCATGGCTCTATTTGCGG-3´	5´ L140H Quickchange
PrfAL140Hrev	5´-CCGCAAATAGAGCCATGCTTCCCGTTAATCG-3´	3´ L140H Quickchange
PrfAG145Cfwd	5´-GGAAGCTTGGCTCTATTTGCTGTCAACTTTTAATCCTGACC-3´	5´ G145C Quickchange
PrfAG145Crev	5´-GGTCAGGATTAAAAGTTGACAGCAAATAGAGCCAAGCTTCC-3´	3´ G145C Quickchange
**Oligonucleotides used for northern blot**		
uphT-U	5´-GCACCACAGAACTAGGAATG-3´	Probe for Northern blot
uhpT-D	5´-CAATATACCCACTGTTGCTTG-3´	Probe for Northern blot
tmRNA-U	5´-CGGCACTTAAATATCTACGAGC-3´	Probe for Northern blot
tmRNA-D	5´-CCTCGTTATCAACGTCAAAGCC-3´	Probe for Northern blot

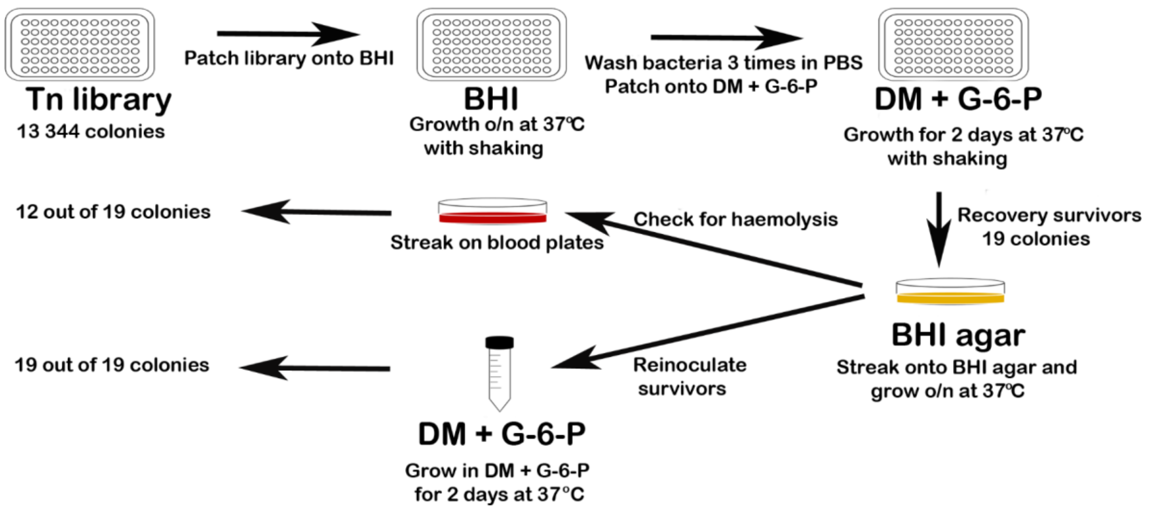

FIG S1 Screening a transposon mutant library for growth in G-6-P. A transposon (Tn) mutant library (n=13344 mutants) were inoculated into 96-well plates with BHI and grown at 37°C with shaking. After growth overnight, the cultures were washed three times in 1 x PBS and then re-inoculated into new 96-well plates containing defined media (DM) supplemented with 0.2% G-6-P as the sole carbon source. Growth was resumed at 37°C with shaking. After two days of growth the cultures were examined for growth. The strains that had the ability to grow in G-6-P where recovered on BHI agar plates. The colonies were re-inoculated into DM with G-6-P in 15 ml tubes and grown at 37°C to verify that the identified mutants still could grow on G-6-P. In addition, the mutants were streaked from the BHI agar plates onto blood agar plates to test for haemolytic activity.

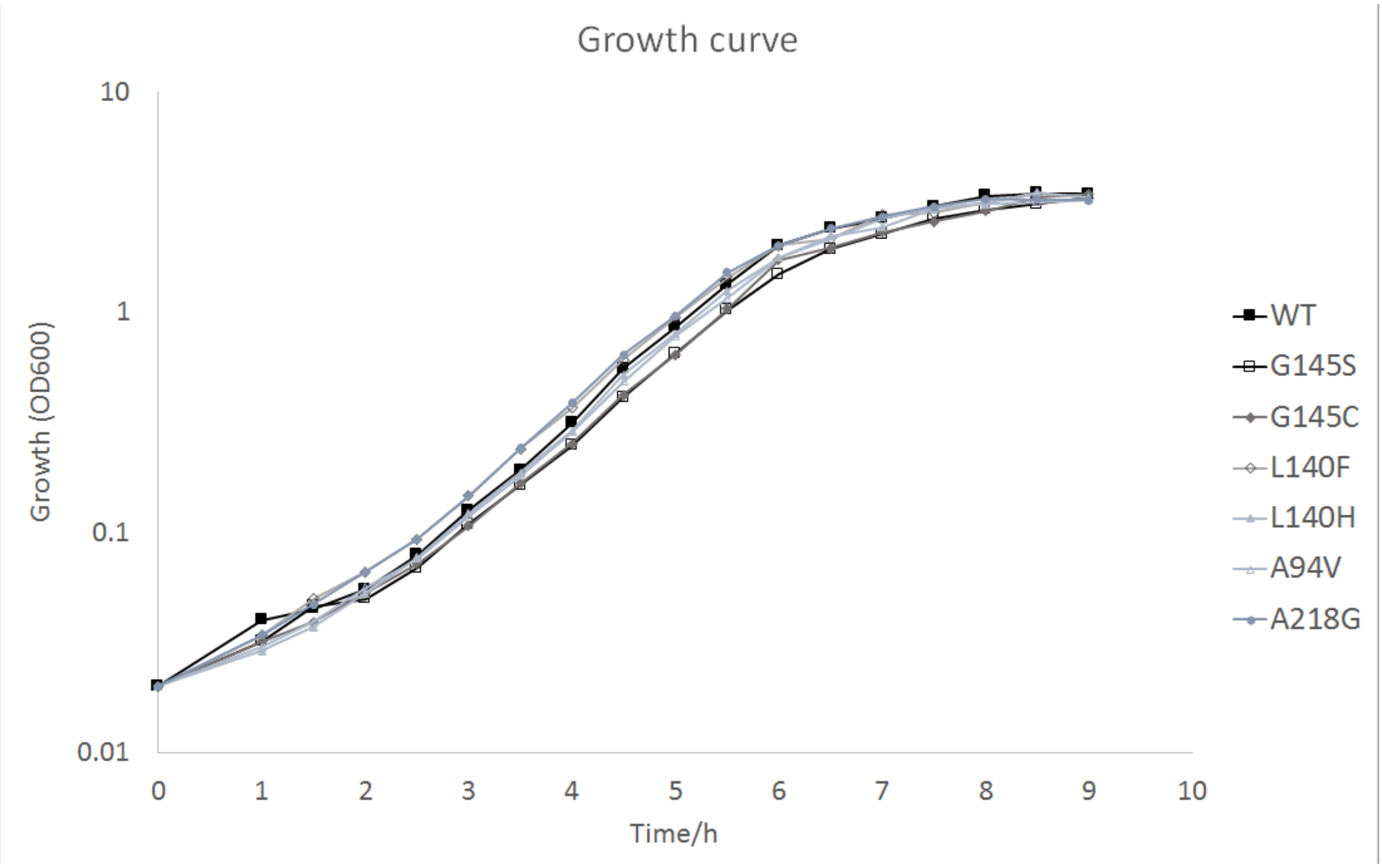

FIG S2 Growth curve for strains carrying point mutations in the PrfA protein. Growth of the indicated strains were tested in BHI at 37°C with shaking. Indicated is a representative of three individual experiments.

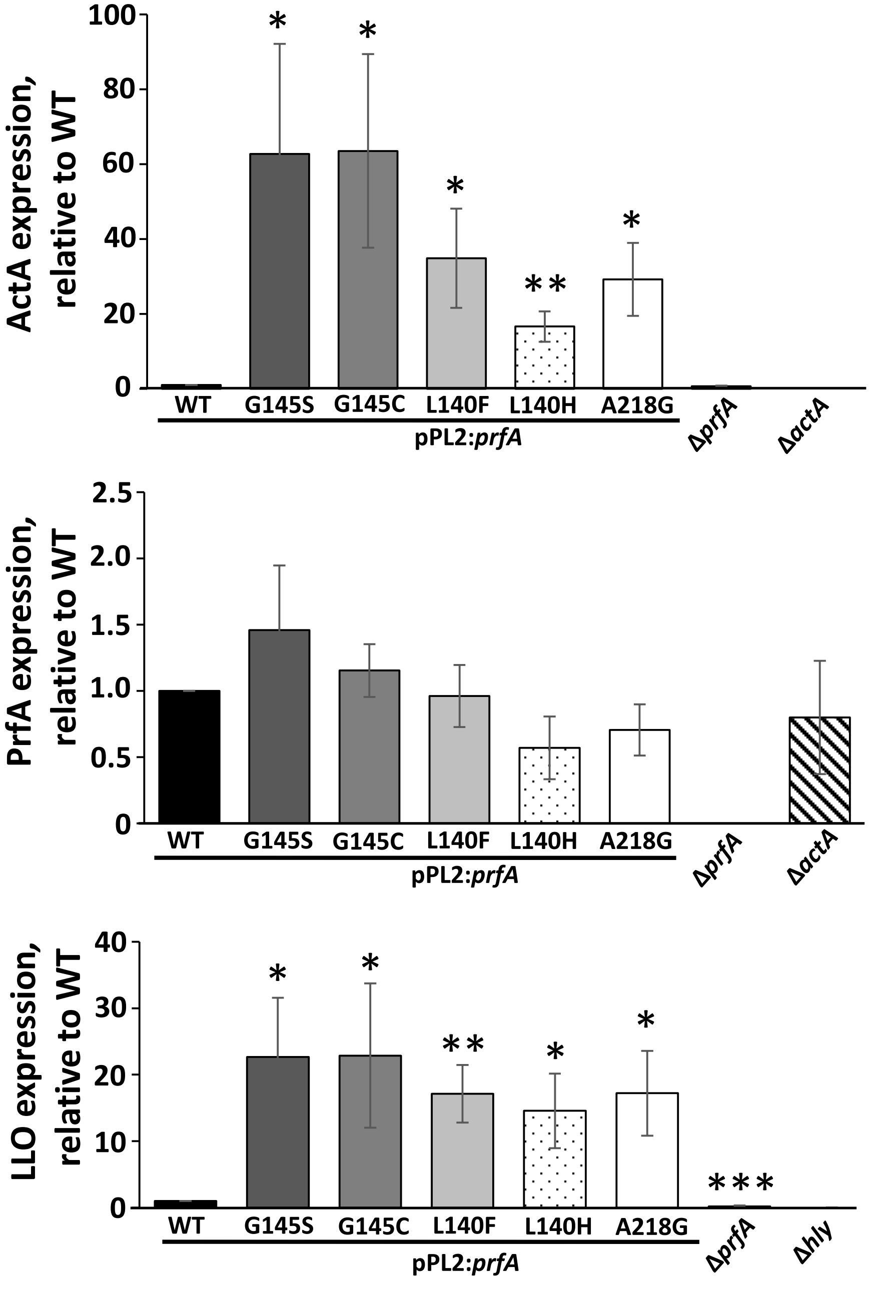

FIG S3 Quantification of ActA, PrfA and LLO expression levels in indicated strains (from Fig. 3A). ActA and PrfA levels were correlated to RpoB whereas LLO levels was correlated to P60. WT was set as 1.0. An average with standard deviation of three independent experiments is shown. Statistical analysis compared infection of the wild type strain with the mutant strains (student's T-test (two-tailed p<0.05*, p<0.01,**, p<0.001,***)).

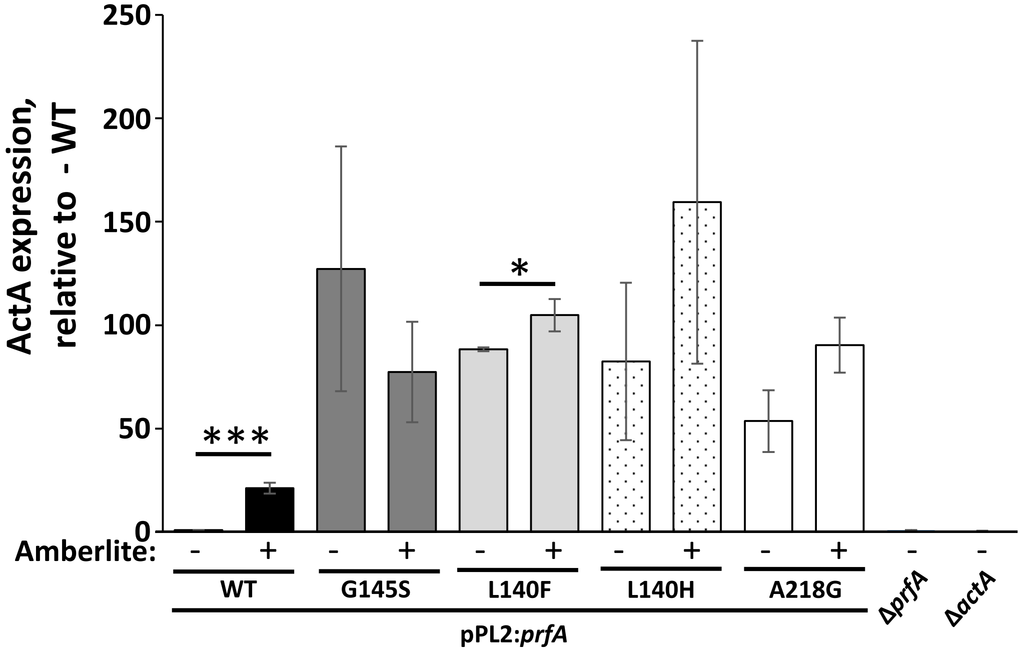

FIG S4 Quantification of ActA levels in indicated strains (from Fig. 3B). ActA levels were correlated to RpoB and WT was set as 1.0. An average with standard deviation of three independent experiments is shown. Statistical analysis compared infection of the wild type strain with the mutant strains (student's T-test (two-tailed p<0.05*, p<0.001,***)).

REFERENCES

1. Good JA, Andersson C, Hansen S, Wall J, Krishnan KS, Begum A, Grundstrom C, Niemiec MS, Vaitkevicius K, Chorell E, Wittung-Stafshede P, Sauer UH, Sauer-Eriksson AE, Almqvist F, Johansson J. 2016. Attenuating Listeria monocytogenes Virulence by Targeting the Regulatory Protein PrfA. Cell Chem Biol 23:404-14.
2. Netterling S, Bareclev C, Vaitkevicius K, Johansson J. 2016. RNA Helicase Important for Listeria monocytogenes Hemolytic Activity and Virulence Factor Expression. Infect Immun 84:67-76.
3. Tiensuu T, Andersson C, Ryden P, Johansson J. 2013. Cycles of light and dark co-ordinate reversible colony differentiation in Listeria monocytogenes. Mol Microbiol 87:909-24.
4. Lauer P, Chow MY, Loessner MJ, Portnoy DA, Calendar R. 2002. Construction, characterization, and use of two Listeria monocytogenes site-specific phage integration vectors. J Bacteriol 184:4177-86.
5. Mengaud J, Dramsi S, Gouin E, Vazquez-Boland JA, Milon G, Cossart P. 1991. Pleiotropic control of Listeria monocytogenes virulence factors by a gene that is autoregulated. Mol Microbiol 5:2273-83.
